# Liposomal bortezomib is active against chronic myeloid leukemia by disrupting the Sp1-BCR/ABL axis

**DOI:** 10.18632/oncotarget.8871

**Published:** 2016-04-20

**Authors:** Xiaojuan Yang, Jiuxia Pang, Na Shen, Fei Yan, Lai-Chu Wu, Aref Al-Kali, Mark R. Litzow, Yong Peng, Robert J. Lee, Shujun Liu

**Affiliations:** ^1^ The Hormel Institute, University of Minnesota, Austin, MN 55912, USA; ^2^ Department of Biological Chemistry and Pharmacology, The Ohio State University, Columbus, OH 43210, USA; ^3^ Division of Pharmaceutics, College of Pharmacy, The Ohio State University, Columbus, OH 43210, USA; ^4^ Division of Hematology, Mayo Clinic, Rochester, MN 55905, USA; ^5^ Department of Thoracic Surgery, State Key Laboratory of Biotherapy, West China Hospital, Sichuan University/Collaborative Innovation Center of Biotherapy, Chengdu, 610041, China

**Keywords:** BCR/ABL, chronic myeloid leukemia, bortezomib, nanoparticle, liposome

## Abstract

The abundance of the BCR/ABL protein critically contributes to CML pathogenesis and drug resistance. However, understanding of molecular mechanisms underlying BCR/ABL gene regulation remains incomplete. While BCR/ABL kinase inhibitors have shown unprecedented efficacy in the clinic, most patients relapse. In this study, we demonstrated that the Sp1 oncogene functions as a positive regulator for BCR/ABL expression. Inactivation of Sp1 by genetic and pharmacological approaches abrogated BCR/ABL expression, leading to suppression of BCR/ABL kinase signaling and CML cell proliferation. Because of potential adverse side effects of bortezomib (BORT) in imatinib-refractory CML patients, we designed a transferrin (Tf)-targeted liposomal formulation (Tf-L-BORT) for BORT delivery. Cellular uptake assays showed that BORT was efficiently delivered into K562 cells, with the highest efficacy obtained in Tf-targeted group. After administered into mice, L-BORT exhibited slower clearance with less toxicity compared to free BORT. Furthermore, L-BORT exposure significantly blocked BCR/ABL kinase activities and sensitized CML cell lines, tumor cells and doxorubicin (DOX) resistant cells to DOX. This occurred through the more pronounced inhibition of BCR/ABL activity by L-BORT and DOX. Collectively, these findings highlight the therapeutic relevance of disrupting BCR/ABL protein expression and strongly support the utilization of L-BORT alone or in combination with DOX to treat CML patients with overexpressing BCR/ABL.

## INTRODUCTION

Chronic myeloid leukemia (CML) is a myeloproliferative neoplasm representing about 15-20% of all cases of adult leukemia in Western populations. The t(9;22) Philadelphia chromosome translocation fuses the BCR gene to the c-ABL proto-oncogene resulting in a chimeric BCR/ABL protein, a constitutively active tyrosine kinase. Aberrant BCR/ABL kinase activity is found in nearly all CML patients [1, 2] and plays a central role in CML pathogenesis [3–5]. Tyrosine kinase inhibitors (TKIs) have demonstrated remarkable clinical efficacy in nearly all chronic phase CML patients [5–7]. However, serious problems arise from TKI therapy, including drug resistance, partial eradication of BCR/ABL-expressing cells and limited effect on the quiescent Ph+ stem cells [8]. TKI resistance in CML is primarily caused by the re-establishment of hyperactive ABL kinase either through acquired mutations in the BCR/ABL kinase domain or its gene amplification [9–11]. In addition, elevated BCR/ABL protein expression is found during the blast crisis phase of CML [12] and in a subset of relapsed CML patients [11, 13]. BCR/ABL expression level also significantly influences the development of BCR/ABL point mutations [12]. These findings, collectively, support the idea that BCR/ABL protein expression could be a promising target for overriding aberrant ABL kinase activity. Thus, to successfully treat resistant CML, alternative strategies are highly desirable to target BCR/ABL expression and its gene regulators with approaches distinct from those employed by conventional tyrosine kinase inhibitors.

Because BCR/ABL promotes proteasome-dependent degradation of tumor suppressor genes (TSGs) [14], bortezomib (BORT), a proteasome inhibitor, was used to block BCR/ABL activity in order to restore TSG expression *in vitro* and *in vivo*. However, in a pilot clinical trial, BORT was found to have minimal efficacy and considerable toxicity in imatinib-refractory CML patients [15]. The suboptimal clinical performance (e.g., significant dose-related toxicity) and the lack of a sufficient therapeutic index calls for new therapeutic approaches that could include different drug targets and/or more efficient delivery vehicles, such as liposomes. Developing a slow-release formulation, such as liposomes, can potentially lead to reduced off-target toxicity and a higher therapeutic index [16].

Liposomes are composed of lipid bilayers and are capable of carrying both hydrophobic drugs. The low production cost, flexibility to carry drugs with various physical chemical properties, low immunogenicity and apparent enhanced drug delivery efficiencies have made liposomes a popular drug delivery carrier. The marketed liposomal drugs, doxorubicin (DOXIL) [17] and daunorubicin (DaunoXome), have also addressed the serious side effects in the form of dose-limiting cardiotoxicity. Further, the specificity of drug delivery can be increased by coating drug-loaded liposomes with ligands targeting specific cell surface markers, like transferrin receptor (TfR), whose expression is increased in CML cells.

In this study, we designed a liposomal formulation of BORT (L-BORT) and its TfR-targeted derivative and characterized its physicochemical properties and ability to deliver the drug to target cells and pharmacokinetic (PK) properties in mice. We assessed its therapeutic potential in CML cell lines, single tumor cells and DOX resistant cells. We demonstrated that BCR/ABL protein expression is positively regulated by Sp1 and in parallel with its kinase activity. Disruption of the Sp1-BCR/ABL axis by *Sp1* siRNA, *miR-29b* and BORT impaired BCR/ABL kinase signaling leading to the blockage of CML cell proliferation. L-BORT achieved enhanced BORT delivery efficiency, improved pharmacokinetic performance, abrogation of Sp1-BCR/ABL function and chemo-sensitization to DOX.

## RESULTS

### Sp1 inhibition by BORT suppresses BCR/ABL kinase signaling

**Figure 1:**
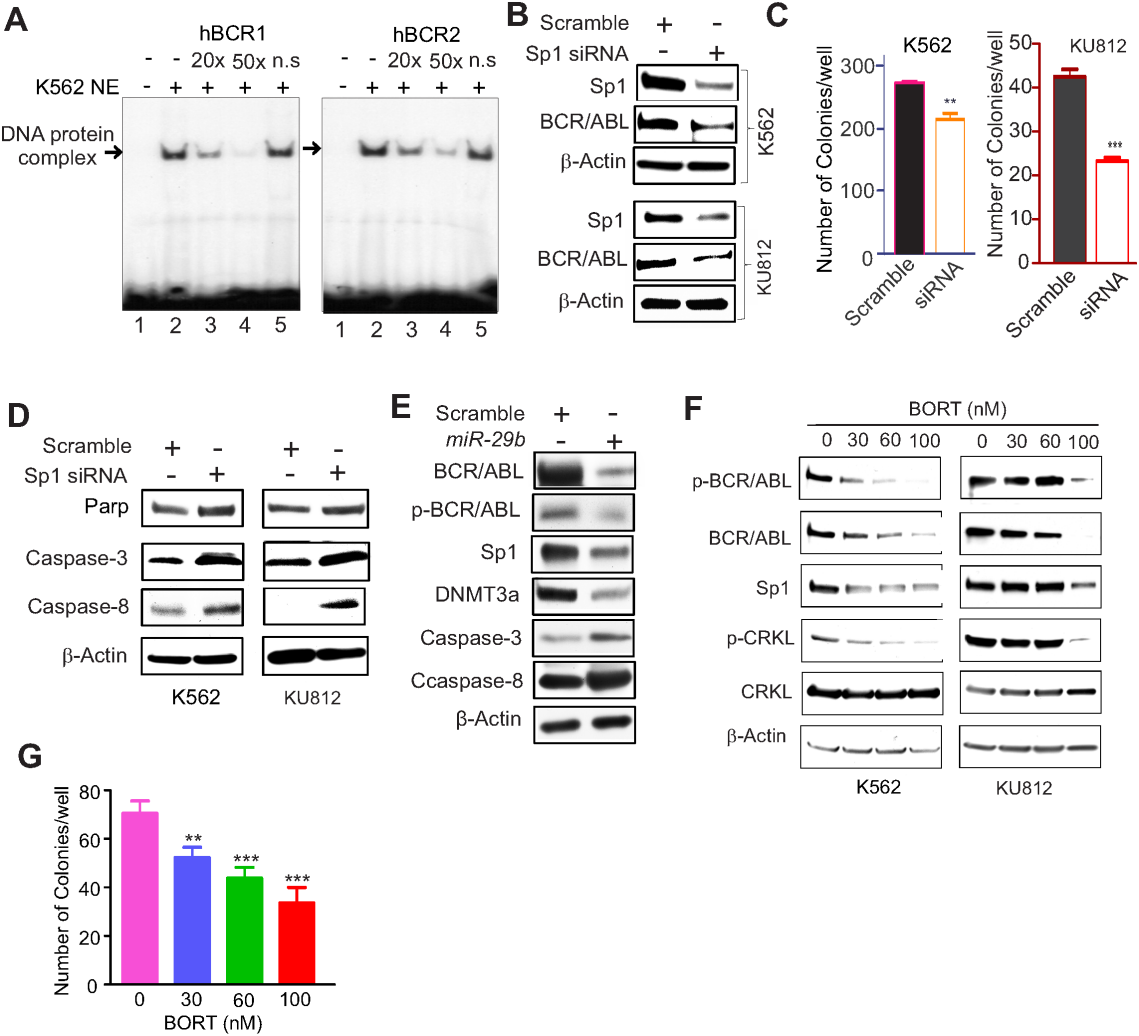
Sp1 inactivation disrupted BCR/ABL signaling **A.** EMSA showing Sp1 binding on *BCR/ABL* promoter. The EMSA probes (hBCR1, hBCR2) covering Sp1 binding sites on *BCR/ABL* gene promoter were labeled by ^32^P and incubated with nuclear extract from K562 cells. The Sp1-DNA protein complex was competed with non-labeled corresponding probes (cold DNA). Note: n.s. cold DNA with TATA site. **B-D.** Modulation of Sp1/*miR29b* network altered BCR/ABL activities. K562 and KU812 cells were transfected with *Sp1* siRNA for 48 hours, and subjected to Western blotting (B, D) or colony-forming assays (C). **E.** K562 cells were transfected with *miR-29b* for 48 hours and the cells were lysed for Western blotting. **F.** K562 and KU812 cells were treated with different doses of BORT for 24 hours and the cells were harvested for Western blotting. **G.** K562 cells were treated with BORT for 6 hours and subjected to colony-forming assays. The data represent three independent experiments; Data are mean ±SD; **p < 0.01, ***p < 0.001.

To elucidate the mechanisms underlying *BCR/ABL* gene expression, we analyzed its promoter region and identified several putative Sp1 binding sites. We performed electrophoretic mobility-shift assays (EMSA) with nuclear extract (NE) prepared from K562 cells and probes (hBCR1 and hBCR2) spanning the *BCR/ABL* promoter regions containing Sp1-binding sites. The ^32^P-lableled hBCR1 and hBCR2 probes yielded slower migrating DNA-protein complexes (Figure 1A, lane 2). The specificity of DNA-protein interactions was demonstrated by competition assays with 20- and 50-fold excess unlabeled *BCR/ABL* promoter probes (cold DNA), in which the unlabeled DNA oligos containing the Sp1-binding sites efficiently and dose-dependently competed away protein binding to both probes (Figure 1A, lanes 3 and 4). In contrast, cold non-specific (n.s.) probes did not significantly impact the formation of Sp1-DNA complex (Figure 1A, lane 5). These data, collectively, suggest a specific interaction between *BCR/ABL* promoter and Sp1 protein.

To determine whether Sp1 enrichment on *BCR/ABL* promoter contributes to *BCR/ABL* expression, we silenced *Sp1* expression in K562 and KU812 cells by transfecting a pool of four siRNAs that targeted different regions of the *Sp1* transcripts. As expected, siRNA-triggered *Sp1* knockdown resulted in reduced BCR/ABL protein expression (Figure 1B), along with impaired clonogenic potential (Figure 1C; K562, scramble 270.5±8.4 versus siRNA 212.3±12.6, **p < 0.01; KU812, scramble 42.3±1.9 versus 23.0±1.1, ***p < 0.001) and the increased activated form of caspases (Figure 1D). Similarly, increased expression of *miR-29b*, a negative regulator for Sp1 [18, 19], suppressed BCR/ABL expression with a concurrent increase of the activated caspases (Figure 1E). The downregulation of DNMT3a was used as a positive control [18]. To pharmacologically inhibit Sp1, we employed BORT that was demonstrated to abolish Sp1 transactivation in our previous studies [19, 20]. As shown in Figure 1F, exposure of K562 and KU812 cells to BORT resulted in inhibition of Sp1 and BCR/ABL protein expression. We also observed that BORT treatment decreased the autophosphorylation of BCR/ABL and the phosphorylation of CRKL, a BCR/ABL downstream effector. Functionally, treatment with BORT significantly disrupted K562 cell colony formation (Figure 1G; 70.3±5.1, 51.8±4.3, 43.5±4.5, 33.3±6.5; **p < 0.01, ***p < 0.001). Together, these results support the idea that Sp1 is a positive regulator for BCR/ABL and that Sp1 inhibition abrogates BCR/ABL kinase signaling.

### Synthesis and validation of L-BORT and Tf-L-BORT

**Figure 2:**
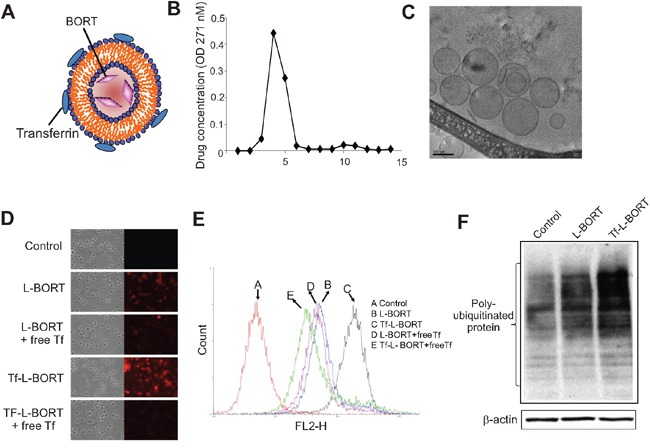
Synthesis and characterization of L-BORT **A.** A model structure of Tf-L-BORT. **B.** Drug entrapment efficiency. L-BORT and free BORT was separated by Sepharose CL-4B column and drug concentration was determined by a Shimadzu UV-visible spectrophotometer. **C.** The Cryo-TEM image of L-BORT. The external dark circle which represents the lipid layer enclosed the internal aqueous volume of the particle. **D-E.** Cellular uptake analysis of L-BORT and Tf-L- BORT. The K562 cells were treated with L-BORT or Tf-L-BORT for 1 hour at 37°C and the cellular uptake of R18-labelled L-BORT was (D) visualized by fluorescence microscope or (E) measured by FACSCalibur flow cytometry. **F.** The K562 cells were treated with L-BORT or Tf-L-BORT for 6 hours and subjected to Western blotting. The data represent three independent experiments.

Because of high plasma protein binding and rapid clearance, the therapeutic index of BORT could be improved. To enhance BORT delivery efficiency, we designed L-BORT and TfR-targeted L-BORT (Tf-L-BORT). A remote-loading method was used to load BORT into liposomes. The lipid composition was HSPC/Chol/PEG_2000_-DSPEat 65/30/5 (mol/mol), intraliposomal buffer was 300 mM meglumine and 300 mM calcium acetate solution (pH 10). Tf-PEG-DSPE was synthesized and incorporated into L-BORT by post-insertion for synthesis of TfR-targeted liposomes. The incorporation of Tf into the liposomes did not change the particle size significantly (not shown). By separating liposomal and free drug using a 10 mL Sepharose CL-4B column, 97.3% or so entrapment efficiencies were achieved and the final BORT content was 0.65 mg/mL, which then could be concentrated by a tangential flow diafiltration method to a higher concentration (Figure 2A and 2B). The colloidal stability of L-BORT was evaluated by monitoring changes in its mean diameter over time. Both L-BORT and Tf-L-BORT remained stable at both 4°C for at least 3 weeks. No significant difference in colloidal stability was observed between L-BORT and Tf-L-BORT. The drug content variation of L-BORT during storage was also tracked and it was found that there was no leakage of entrapped BORT over 50 days of storage.

To further study the morphology of L-BORT before Tf incorporation, L-BORT was first prepared in a controlled environment vitrification system (CEVS) and followed by Philips CM120 YEM microscope with Gatan 791 MultiScan CCD camera for Cryo-TEM imaging. As shown in Figure 2C, this Cryo-TEM image revealed spherical particle morphology with diameter around 100 nm, which verified the data obtained by dynamic light scattering. The external dark circle represents the lipid layer.

Cellular uptake of R18-labelled L-BORT was evaluated in K562 cells which highly expressed TfR (not shown). Under fluorescence microscopy, we found that Tf-L-BORT was efficiently internalized by the cells after 1-hour of incubation and the level of uptake was much higher than that of L-BORT (Figure 2D and 2E). Co-incubation of cells with free Tf significantly reduced cellular uptake of Tf-L-BORT, but did not affect the cellular uptake of L-BORT, suggesting that the enhancement of BORT cellular uptake via Tf-L was due to the presence of Tf ligands on the liposome surface. The TfR-mediated cellular uptake of BORT through Tf-L-BORT was quantified by flow cytometry. It was also found that the cellular uptake of both L-BORT and Tf-L-BORT was concentration dependent (not shown). Compared to L-BORT, the higher efficacy of BORT delivery by TfR-targeted nanoparticles was verified by the greater accumulation of poly-ubiquitinated proteins in the presence of Tf-L-BORT (Figure 2F).

### Pharmacokinetics of L-BORT *in vitro* and *in vivo*

**Figure 3:**
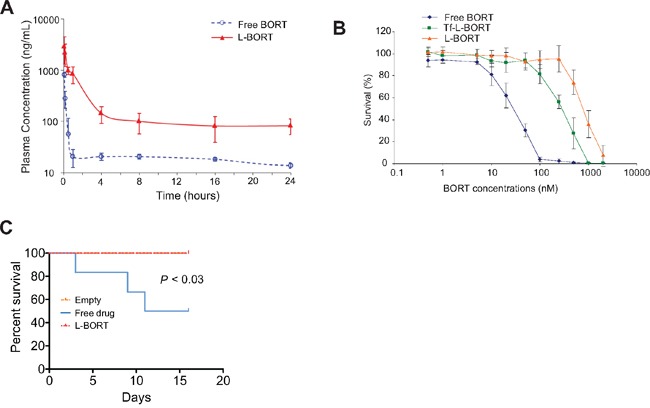
L-BORT has decreased clearance and reduced cytotoxicity **A.** Pharmacokinetic of L-BORT. ICR mice (n=5 mice/group) received intravenous injections of BORT at 1.0 mg/kg via tail vein. Plasma clearance rates of free- and L-BORT were compared and shown by the plasma BORT concentration –time plot. **B.** The K562 cells were treated with free-, L- or Tf-L-BORT for 48 hours and the cell proliferation was assessed by MTT assays. The results are the mean of 6 repeats. Error bars are standard deviations. **C.** Toxicity assays *in vivo*. Free- or L-BORT was administered to BALB/cByJ normal mice (n=6 mice/group) through the tail-vein at 2.5gm/kg every four days. Mice treated with equal volume of sterile saline were used as controls.

BORT exposure and elimination in mice were investigated following a tail vein injection of L-BORT at 1.0 mg/kg dose. The PK of free BORT at the same dose level was assessed in parallel as a comparison. The plasma concentration-time plot (Figure 3A) showed that L-BORT had a much higher drug exposure and slower elimination rate than the free BORT. PK parameters were obtained by Non-compartmental PK analysis in Phoenix WinNonlin. As shown in Table 1, the blood circulation half-life of BORT in the liposome formulation elongated from 12.4 hours to 23.5 hours when compared to the free BORT, resulting in the high plasma exposure (AUC) of 6.56 μg·hr/mL, which was about 10 fold of that of free BORT (0.469 μg·hr/mL). Also the clearance was reduced from 1510 mL/hr/kg to 153 mL/hr/kg, suggesting that using liposome as carriers for BORT is potentially beneficial to therapeutic efficacy.

**Table 1:** The pharmacokinetic parameters of free- and L-BORT

PK Parameter^a^^b^/ Formulation	C_max_ (μg/mL)	AUC_inf_ (μg·hr/mL)	t_1/2_ (hr)	CL (L/hr/kg)	V (L/kg)
Free BORT	0.758 (5.93%)	0.661 (12.8%)	12.4 (15.5%)	1.51 (17.9%)	27.1 (17.6%)
L-BORT	2.58 (14.2%)	6.56 (20.1%)	23.5 (14.6%)	0.153 (16.3%)	5.18 (15.1%)

a Data represent the mean (%CV) (n=5).

b PK parameter C_max_ was the maximum plasma concentration, AUC_inf_ was area under plasma concentration-time curve extrapolated to time of infinity, t_1/2_ was the elimination half- life, CL was total body clearance, and V was volume of distribution.

### Toxicity evaluation of L-BORT *in vitro* and *in vivo*

Despite impressive therapeutic efficacy, BORT also induces severe non-specific toxicities through binding to serum proteins. To investigate if the entrapment of BORT in liposomes can decrease its side effects while maintaining higher delivery efficiency, we incubated the drug formulation with K562 cells for 48 hours and then measured the cell survival rates by MTS assays. As shown in Figure 3B and Table 2, L-BORT suppressed cell proliferation with an IC_50_ of 814 nM (*p* < 0.05). Increased cytotoxicity was induced by Tf-L-BORT (IC_50_ 305 nM) compared to that by L-BORT, which was probably caused by the enhanced cellular uptake of the former.

**Table 2:** Cytotoxicity of L-BORT to K562 cells

BORT formulation	IC_50_ of BORT (nM)^a^
Free BORT	39.3 ± 2.27
L-BORT	814 ± 21.4^b^
Tf-L-BORT	305 ± 19.5^b^

a Data represent the mean ±SD (n=6).

b Statistically significance vs all other groups (*p* < 0.05).

To determine the toxicity *in vivo*, liposomal vehicle, free or L-BORT was administrated through the tail vein to normal BALB/cByJ mice (6-8 weeks old, 22 ± 5 g) in a small volume (0.2 ml) at normal pressure. The dose used was 2.5 mg/kg every four days. We observed that 50% of mice with free drug died in two weeks, but no observable toxicity was observed in L-BORT-treated mice (Figure 3C). These results indicate that formulated BORT had much lower toxicity.

### L-BORT impairs BCR/ABL activity and inhibits cell proliferation

**Figure 4:**
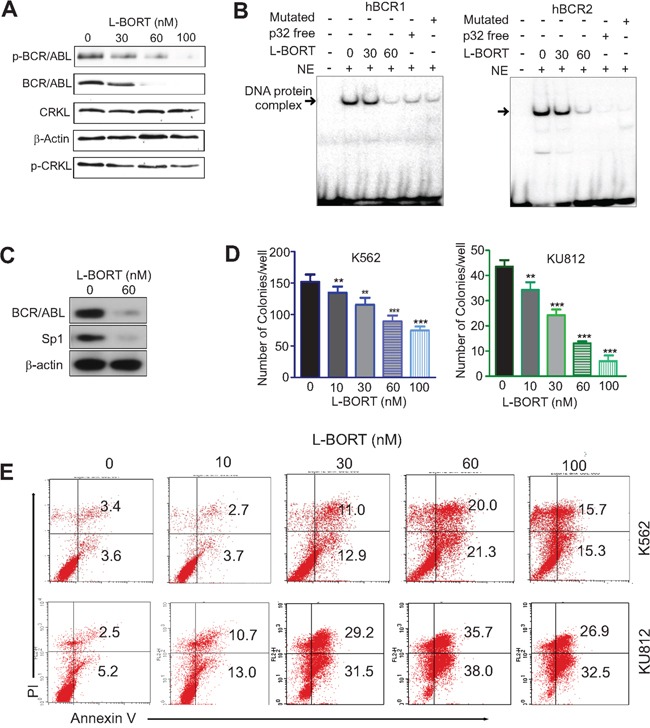
L-BORT treatment impairs BCR/ABL signaling and cell proliferation **A.** Western blotting in K562 cells exposed to L-BORT for 48 hours. **B.** K562 cells were treated with L-BORT for 24 hours and subjected to EMSA assays for Sp1 binding in *BCR/ABL* promoter using ^32^P-labled hBCR1/hBCR2 probes. NE, Nuclear extract. **C.** Western blot in K562 cells treated with L-BORT for 24 hours. D, The K562 and KU812 cells were treated with L-BORT or Tf-L-BORT for 6 hours and subjected to colony-forming assays. **E.** Flow cytometry assays for cell apoptosis in K562 and KU812 cells treated with L-BORT for 48 hours. The data represent three independent experiments; Data are mean ±SD; **p < 0.01, ***p < 0.001.

To determine the inhibitory effect of L-BORT on BCR/ABL kinase activity, K562 cells were treated with various doses of L-BORT for 48 hours followed by Western blot to assess BCR/ABL protein levels and its activity. Similar to free BORT, L-BORT significantly decreased the expression of BCR/ABL and the phosphorylation of BCR/ABL and CRKL (Figure 4A). Mechanistic investigations showed that Sp1-DNA complex in BCR/ABL promoter was disrupted upon exposure to L-BORT (Figure 4B). The abolishment of Sp1-DNA complex by hBCR1/hBCR2 probes without ^32^P-label or with mutated Sp1-binding sites demonstrated the Sp1 binding specificity in BCR/ABL promoter. Given that L-BORT impaired Sp1 protein expression (Figure 4C), these results suggested that L-BORT-mediated BCR/ABL dysfunction occurs through inhibition of Sp1-associated transcription. Functionally, L-BORT treatment in K562 and KU812 cells dose-dependently disrupted their clonogenic ability (Figure 4D; K562, 151.0±11.8, 133.8±9.8, 114.8±11.0, 88.5±9.2, 73.8±6.3; KU812, 43.3±2.6, 34.0±3.1, 24.0±2.2, 12.8±0.8, 5.8±1.6; **p < 0.01, ***p < 0.001) and promoted cell apoptosis (Figure 4E). These data support the therapeutic potential of L-BORT in CML.

### Subtoxic L-BORT potentiates sensitivity of doxorubicin resistant K562 cells to doxorubicin

**Figure 5:**
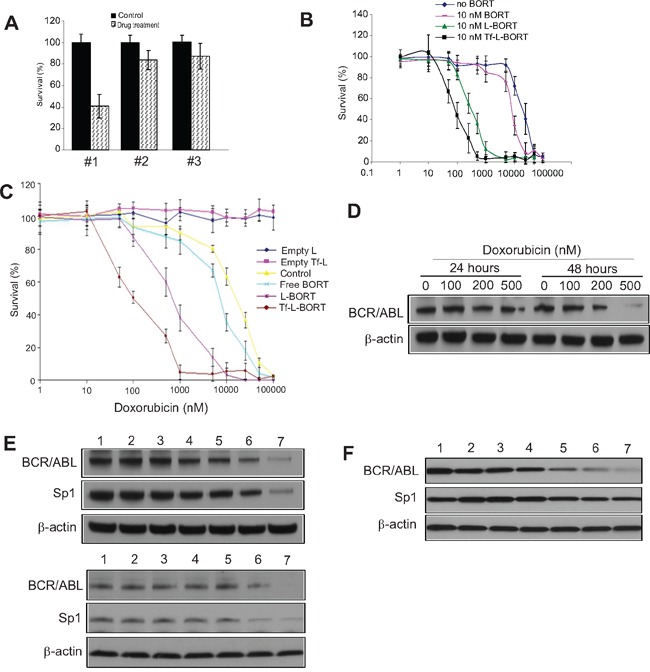
Subtoxic L-BORT potentiates sensitivity of BCR/ABL positive cells to DOX **A.** K562 cells were treated with (#1) 100 nM DOX for 24 hours first, then 2 nM L-BORT for further 24 hours; (#2) 2 nM L-BORT for 24 hours first, then 100 nM DOX for further 24 hours; (#3) 10 nM L-BORT and 100 nM DOX together. The cell proliferation was determined by MTT assays. **B.** K562 cells were treated with indicated doses of DOX plus free-, L- or Tf-L-BORT for 48 hours and MTT assays were used to measure cell proliferation. **C.** K562 single tumor cells were treated with indicated doses of doxorubicin plus free-, L- or Tf-L-BORT for 48 hours and the cell proliferation was determined by MTT assays. **D.** Western blotting in K562 cells treated with different doses of doxorubicin. **E.** Western blotting in K562 (upper) and KU812 (lower) cells treated with suboptimal doses of L-BORT or/and DOX for 48 hours. **F.** Western blotting in K562 single tumor cells treated with DOX or/and L-BORT for 48 hours. In A-C, the results are the mean of 6 repeats representing two independent experiments. Error bars are standard deviations; In D-F, The data represent three independent experiments; In E and F, 1, Empty Tf-Lip; 2, Empty Lip; 3, L-BORT 10 nM; 4, Tf-L-BORT 10 nM; 5, DOX 200 nM; 6, L-BORT 10 nM + DOX 200 nM; 7, Tf-L-BORT 10 nM + DOX 200 nM.

To determine if L-BORT and Tf-L-BORT could enhance the cytotoxicity of agents used for CML therapy, we employed the MTS assay to evaluate the cytotoxic effects of doxorubicin, alone and in combination with a subtoxic BORT formulation at 2 nM to doxorubicin resistant K562/DOX cells. Initially, we determined the effects of drug treatment sequences on chemosensitivities by treating the K562/DOX cells with 100 nM doxorubicin for 24-hour before, after or simultaneous addition of subtoxic 2 or 10 nM Tf-L-BORTs followed by another 24 hour co-incubation. Only with the treatment order of doxorubicin first and then Tf-L-BORT, K562/DOX cells showed significant sensitivity to doxorubicin with as low as 41.4% survival rate at doxorubicin concentration of 100 nM (Figure 5A, #1). Meanwhile, the other two treatment methods as well as 2 nM Tf-L-BORT did not induce significant cell death (Figure 5A, #2 and #3). We then applied this treatment strategy to K562/DOX cells exposed to various concentrations of doxorubicin. As shown in Figure 5B and Table 3, L-BORT enhanced the K562/DOX sensitivity to doxorubicin with IC_50_ decreasing from 8 μM to 300 nM, compared to free BORT (*p* < 0.05). Tf-L-BORT further increased the K562/DOX chemosensitivity to doxorubicin, shown by the decreased IC_50_ to 90 nM compared to that in the absence of L-BORT (*p* < 0.05). The survival rate of K562/DOX cells after 15 μM doxorubicin treatment combined with subtoxic Tf-L-BORT was 7 times lower than that with same amount of L-BORT (*p* < 0.05). Therefore, Tf-L-BORT was more effective in chemosensitization than L-BORT.

**Table 3:** Sensitizing of K562/DOX cells by L-BORT to DOX

BORT formulation	IC_50_ of DOX (μM)^a^
No BORT	20.2 ± 8.75
Free BORT	8.14 ± 0.549
L-BORT	0.318 ± 0.0182^b^
Tf-L-BORT	0.0906 ± 0.0182^b^

a Data represent the mean ±SD (n=6).

b Statistically significance vs all other groups (*p* < 0.05).

### L-BORT sensitizes single tumor cells to DOX and synergizes with DOX to downregulate BCR/ABL protein expression

To investigate how L-BORT influences tumor cell growth, tumors were harvested from K562 tumor bearing mice and separated into single cells by collagenase. The single tumor cells were then cultured in RPMI1640 medium with 10% FBS and the cell survival was then evaluated in the presence of doxorubicin combined with subtoxic BORT. As shown in Figure 5C and Table 4, a synergistic effect of toxicity to single tumor cells was found between DOX and free BORT, consistent with what we have observed in K562 cells. IC_50_ of DOX to single tumor cells decreased from 17 μM to 2.3 μM with help of free BORT. L-BORT, with enhanced BORT delivery, amplified the synergy effect to approximately a 70-fold increase in chemosensitivity (*p* < 0.05). Tf-L-BORT was more effective in chemosensitizing tumor single cells than L-BORT, with decreased IC_50_ of doxorubicin at 104 nM.

**Table 4:** Sensitizing of K562 single tumor cells by L-BORT to DOX

BORT formulation	IC_50_ of DOX (μM)^a^
no BORT	17.1 ± 0.534
free BORT	2.31 ± 0.755
L-BORT	0.712 ± 0.113
Tf-L-BORT	0.104 ± 0.0271

a Data represent the mean ±SD (n=6).

^b^ Statistically significance vs all other groups (*p* < 0.05).

Having demonstrated that L-BORT and DOX synergistically inhibited CML cell proliferation, next we sought to determine the underlying mechanisms. Initially, K562 cells were treated with different doses of DOX. We found that doxorubicin dose-dependently downregulated BCR/ABL expression at 48 hours (Figure 5D). Based on this finding, we employed 200 nM DOX and treated K562 cells with DOX alone or in the combination with different BORT formulations. As expected, combination of DOX with Tf-L-BORT induced the most suppressed expression of BCR/ABL and Sp1 compared to other groups (Figure 5E, upper), which was confirmed in KU812 cells with similar treatment (Figure 5E, lower). Importantly, in comparing the exposure of K562 single tumor cell to DOX alone or plus BORT formulations, the combined Tf-L-BORT and DOX resulted in the most downregulation of BCR/ABL and Sp1 (Figure 5F). These results support the idea that L-BORT sensitizes CML cells to DOX through more pronounced inhibition of Sp1-BCR/ABL axis.

## DISCUSSION

It is well known that BCR/ABL expression levels play a critical role in its kinase activity, CML disease progression, drug resistance and the development of point mutations [9, 12, 21, 22]]. However, it remains largely unknown how the BCR/ABL gene is regulated. While inhibitors targeting BCR/ABL kinase activity have been broadly utilized in clinical trials with great success, the outcomes are not yet optimal. In this study, we demonstrated that Sp1 is a positive regulator of the BCR/ABL gene, shedding light on how BCR/ABL is overexpressed in CML. We present evidence that Sp1 dysfunction leads to the blockage of BCR/ABL signaling and CML cell proliferation, thus identifying the Sp1-BCR/ABL axis as a promising target for overcoming aberrant BCR/ABL activity. We showed that L-BORT has better pharmacokinetics and pharmacodynamics with less cytotoxicity *in vitro* and *in vivo*, opening a therapeutic window for the application of Sp1 inhibitors, including BORT, in CML subpopulations characterized by BCR/ABL overexpression.

Sp1 is a zinc finger transcriptional factor. Our previous investigations demonstrated that Sp1 is significantly involved in the regulation of DNA methyltransferases [18, 20], RTKs (KIT, FLT3) [19, 23] and *miR-29b* [19]. We showed that Sp1 specifically binds to the BCR/ABL gene promoter, and genetic and pharmacologic inactivation of Sp1 disrupted BCR/ABL protein expression and phosphorylation. These findings unveil BCR/ABL as an innovative member of Sp1 targeted family. Given the crucial role of Sp1 in BCR/ABL kinase activity, we employed BORT, a well-established Sp1 inhibitor [19, 20], and found that exposure of CML cells to BORT resulted in impairment of the Sp1-BCR/ABL axis and subsequent suppression of CML cell proliferation. These results identified the Sp1-BCR/ABL axis as a new molecular regulator underlying the anti-cancer actions of BORT and are in line with our previous discoveries in AML [19, 20, 24] showing that BORT treatment disrupts Sp1-dependent KIT/FLT3 kinase signaling, DNA hypermethylation and restores *miR-29b* expression. Given that BORT was also found to increase TSG expression that is silenced by BCR/ABL-promoted proteasome-dependent degradation [14], further investigations are necessary to clarify the role of BORT in controlling CML growth.

Although ours and other studies show that BORT efficiently inhibits survival and induces apoptosis in BCR/ABL cells, a pilot study [15] reported minimal efficacy and considerable toxicity in CML patients receiving BORT therapy. While it remains to be further elucidated, such disappointing outcomes may result from the inefficiency and non-specificity of BORT delivery *in vivo*. Therefore, we developed a liposomal formulation of BORT with the objective to lower non-specific toxicity while maintain high efficacy to sensitize cancer cells to typical chemotherapy drugs, like DOX. In addition, liposomes with targeting ligands, such as antibodies, growth factors, Tf and vitamin folate, can decrease the serum aggregation and improve the specific drug delivery to diseased cells for different reasons. Besides the shielding effect and specificity, liposomes containing multiple targeting ligands have multivalent binding to specific cell surfaces and therefore can deliver a larger payload of drugs than liposomes without targeting ligands. Correspondingly, more and more receptor overexpression has been found in tumors and other disease tissues. For example, the folate receptor (FR) is found to be up-regulated in more than 90% of non-mucinous ovarian carcinomas while its expression in normal tissues is highly restricted. Additionally, the amplification of human epidermal growth factor receptor-2 (HER2) gene has been observed in breast cancer and ovarian cancers. The liposomes with targeting ligands to these receptors have been identified to be efficient and specific to deliver drugs *in vitro*. In the current study, Tf-attached liposomes (Tf-L) were formulated for the enhanced BORT delivery to K562 cells.

The establishment of the L-BORT preparation procedure was based on the optimization of a remote drug loading method and intraliposomal buffer system, for the objective of high drug entrapment efficiency. When intraliposomal buffer of meglumine and calcium acetate solution was replaced by a sorbitol solution (pH 8), neither remote loading method nor passive entrapment method could bring drug entrapment efficiency to higher than 15%. By comparing various methods, it was found that our method herein had the highest drug entrapment (97.3%) and appropriate particle size (~100 nm) for enhanced permeability and retention (EPR) effect. It was therefore finally adopted for the following *in vitro* efficacy and *in vivo* PK studies.

The cellular uptake experiments showed that TfR-adjusted BORT uptake was much higher than that by non-targeted delivery and the subtoxic Tf-L-BORT treated K562/DOX also obtained the enhanced chemosensitivity to anticancer drug, DOX, indicating the improved but consistent efficacy compared to free BORT. Although there is high concentration of free Tfs existing in circulation, we do not think they can compete with Tf-L-BORT to bind TfR on cell surface, because most of them are not iron-loaded and, therefore, have lower affinity for the TfR than the holo-Tf used in the Tf-L-BORT. In fact, gallium citrate, a clinical radiopharmaceutical, acts by *in vivo* loading on to circulating Tf and is able to effectively target tumors in patients despite the presence of an abundance of the unloaded Tf in circulation. In addition, Tf-L-BORT contains multiple Tfs on each particle and is capable of multivalent interaction with the target cells. Such interaction is likely to result in a great increase in affinity compared to the Tf found in circulation, which is monovalent. Therefore, the Tf-L-BORT should be able to out-compete endogenous Tf found in circulation for TfR binding.

L-BORT had less toxicity than free BORT with approximately 20-time and 10-time increases of IC_50_ from 39 nM to 814 nM and 305 nM, respectively, for L-BORT and Tf-L-BORT, which could probably be explained by the hypothesis of slow release of L-BORT following the cellular uptake via endocytosis. These results identified the advantage of liposome, especially Tf-liposome, as drug delivery system to efficiently and specifically deliver BORT to targets and could be helpful to decrease the side effects for treating leukemia patients. Pretreatment of K562/DOX cells with subtoxic Tf-L-BORT reversed the drug resistance to DOX. Pharmacokinetic study demonstrated that L-BORT had prolonged a blood circulation time and decreased clearance compared to the free drug. Consistent with this are the findings that, as compared to free BORT, L-BORT provides long-term disease free survival in mice bearing large granular lymphocyte leukemia without any evidence of toxicity [25]. Thus, switching to a liposomal formulation will offer the potential to alter the pharmacokinetics and provide a more favorable efficacy/toxicity profile for BORT in human trials.

## MATERIALS AND METHODS

### Cell lines and compounds

Cell lines (K562 and KU812) were obtained from American Type Culture Collection (Manassas, VA, USA). BORT was obtained from LC Laboratories (Woburn, MA). On-targetplus Smart pool SiRNA for *Sp1* gene, *miR-29b* and their corresponding scrambles were purchased from Thermo Fisher Scientific (Waltham, MA).

### Transient transfection and western blot

*Sp1* siRNA, *miR-29b* and their scrambles were introduced into K562 cells by Lipofectamine™ 2000 (Invitrogen) and the Western blotting was performed as previously described [20, 26, 27]. The antibodies used are: Sp1, DNMT3a, β-actin and ubiquitin (Santa Cruz Biotechnology, Santa Cruz, CA); ABL, phospho-ABL, caspase-3 and caspase-8 (Cell Signaling Technology, Danvers, MA); Parp, CRKL and p-CRKL (Millipore, Billerica, MA).

### Electrophoretic mobility-shift assays (EMSA)

EMSA with nuclear extracts and ^32^p-labeled probes were performed as previously described [19, 20]. The sequences of the oligoes for BCR/ABL promoter probes are listed in supplementary material.

### Clonogenic assays

Methylcellulose colony-forming assays were carried out in MethoCult® mixture (Stem Cell Technologies Inc., Vancouver, Canada) as previously described [26, 27]. Briefly, six hours after transfection or exposure to drugs, the cells were harvested and diluted with IMDM + 2% FBS. About 1,000 cells were distributed into 6-well plate. The colonies were scored in 7-10 days.

### Preparation and characterization of L-BORT and Tf-L-BORT

The synthesis of L-BORT was done as previously described [25]. Briefly, a remote loading method was used to prepare L-BORT. Liposomes were initially synthesized encapsulating a “drug-trapping” solution of meglumine and calcium acetate. External buffer was removed and BORT was then added to initiate drug loading. Finally, unencapsulated BORT was removed by gel filtration. Tf ligand was incorporated into BORT-loaded liposomes by a post-insertion method and the resulting L-BORT and Tf-L-BORT were sterilized by filtration through a 0.22 μm membrane filter. Liposome size distribution was determined by dynamic light scattering on a NICOMP Submicron Particle Sizer Model 370 (NICOMP, Santa Barbara, CA). To determine the drug content, the liposomes were lysed by methanol and the BORT concentration in the lysate was determined by absorption at 271 nm on a Shimadzu UV-visible spectrophotometer. Loading efficiency of BORT in liposomes was calculated based on the ratio of the amount of free and liposomal drugs, which were separated by the Sepharose CL-4B column.

### Measurement of cellular uptake and evaluation of cytotoxicity

Cellular uptake of R18-labeled Tf-L-BORT or L-BORT was evaluated in K562 cells. About 4×10^5^ cells were incubated with liposomes at 37°C. After 1-hour of incubation, the cells were then washed three times with PBS, photographed on a Nikon fluorescence microscope (Nikon, Küsnacht, Switzerland) and measured by a FACS Calibur flow cytometry (Becton Dickinson, Franklin Lakes, NJ). To evaluate BORT toxicity, K562 cells were treated by different concentration of free- or L-BORT. Regarding to the chemosensitivity to doxorubicin, the K562/ DOX cells were pre-treated with doxorubicin for 24 hours and then subtoxic BORT formulations were added for additional 24 hours. Cellular viability was assessed by MTT proliferation assays (Promega, Madison, WI).

### Pharmacokinetics of L-BORT

BORT (1.0 mg/kg) was injected into ICR mice via the tail vein and blood samples were collected at various time points. Plasma was isolated by centrifugation at 1500×g for 10 minutes and stored at −20°C. Concentration of BORT in the plasma was calculated from boron concentration the sample, which was measured by coupled plasma-optical emission spectrometers (ICP-OES). For ICP-OES measurement, the plasma samples were prepared as previously described [28]. Data analysis and PK parameter calculations were carried out in Phoenix® WinNonlin® 6.3 (Certara, Princeton, NJ).

### Preparation of single tumor cells

K562 cells (10×10^6^) were subcutaneously injected into the flank of 4-6 week old NOD/SCID mice (The Jackson Laboratory, Bar Harbor, ME). When the tumor size approached 1000 mm^3^, the tumors were harvested from mouse flank and cut into pieces in PBS buffer. Collagenase was added into the above buffer and incubated for 2 hours at 37°C with shaking. The digested tumor cell suspension was filtered through a cell strainer and centrifuged at 1200 rpm for 5 minutes. The separated tumor cells were kept in cell frozen medium and stored in liquid N_2_ until use. All animal studies were performed in accordance with the institutional guidelines for animal care and under the protocols approved by the Institutional Animal Care and Use Committee University of Minnesota.

### Statistical analysis

Data were generally represented as mean ±standard deviations (SD). Comparisons between groups (control versus treatment) were made by 2-tailed Student's t-tests using the MiniTAB software (Minitab Inc., State College, PA) or ANOVA analysis where applicable. *P* < 0.05 was used as the cutoff for defining statistically significant differences.

## SUPPLEMENTARY MATERIALS AND METHODS


