# Supplementary material for: Liposomal bortezomib is active against chronic myeloid leukemia by disrupting the Sp1-BCR/ABL axis
Source: Oncotarget. 2016 Apr 20;7(24):36382–94. doi: 10.18632/oncotarget.8871 (PMC5095007; doi:10.18632/oncotarget.8871)
Supplement: Supplementary file 1 [file oncotarget-07-36382-s001.pdf]

Liposomal bortezomib is active against chronic myeloid leukemia by disrupting the Sp1-BCR/ABL axis

SUPPLEMENTARY MATERIALS AND METHODS

Material and methods

Hydrogenated soy phosphatidylcholine (HSPC), cholesterol (Chol) and distearoyl phosphatidylethanolamine-N-[maleimide-polyethylene glycol, MW 2000] (Mal-PEG2000-DSPE) were purchased from Avanti Polar Lipids (Alabaster, AL). Methoxy-PEG2000-DSPE (PEG2000-DSPE) was purchased from Genzyme Corporation (Cambridge, MA). Human holo-transferrin (Tf), 2-iminothiolane (Traut's reagent) and other chemicals were purchased from Sigma-Altrich Chemical Co. (St. Louis, MO). Octadecylrhodamine B chloride (R18) was obtained from Molecular Probes (Eugene, OR, USA).

Cell culture

Leukemia cells K562, KU812 and DOX-resistant K562 cells (K562/DOX) were cultured in RPMI 1640 media supplemented with 10% fetal bovine serum (FBS) (Invitrogen) plus 100 U/mL penicillin and 100 µg/mL streptomycin at 37°C in a humidified atmosphere containing 5% CO_2. For measuring the DOX cytotoxicity, K562/DOX was incubated in medium with 0.1 µM DOX and the DOX was washed out 3 days before the drug treatment.

Cell proliferation and apoptosis assays

MTT cell proliferation assays were performed according to manufacturer's instruction (Promega, Madison, WI). Briefly, ~10,000 K562, KU812 or K562/DOX cells in 100µl RPMI-1640 medium supplemented with 10% of FBS was dispensed into 96-well flat-bottomed microplates and drugs were added after 24 hours of incubation at 37 °C in humidified atmosphere with 5% CO_2. The cells were cultured for another 24 or 48 hours, and 20µl of MTT reagent was added to each well. The microplates were incubated at 37 °C for another 2~4 hours. Absorbance was read at 490 nm using Multiskan Spectrum microplate reader (Thermo Electron Corporation, Waltham, MA), and the results were expressed as a ratio of the treated cells over the untreated cells (as 100%). Three wells were sampled per each experimental group in a given experiment. Averages were reported ±Standard Deviation. Cell apoptosis assays were performed using Annexin V-PE Apoptosis Detection Kit (BD PharmingenTM, San Diego, CA) according to the manufacturer's instruction, and followed by flow cytometry analysis.

Preparation of non-targeted and TfR-targeted L-BORT

A remote loading technology has been developed to prepare L-BORT. In details, a mixture of HSPC, Chol and PEG2000-DSPE at a molar ratio of 65/30/5 was dissolved in the chloroform and 0.1 mol of R18 was added to the formulations for uptake studies. A thin film of lipids was created by rotary evaporation under a vacuum, and then the resultant film was rehydrated in the buffer containing 300 mM meglumine and 300 mM calcium acetate solution (pH 10) to form empty liposomes with lipid concentration at 50 mg/mL. The liposomes were repeatedly freeze-thawed and extruded under pressure through 100 nm membranes at 55 °C for 3 times. The outer buffer is then exchanged to HEPES-buffered saline (HBS, 20 mM HEPES, 145 mM NaCl, pH 6.5) by gel-filtration through Sepharose CL-4B column, resulting empty liposomes ready for drug loading. BORT was dissolved in a very small volume of DMSO and added into the above empty liposome suspension at final drug concentration of 0.65 mg/ml, and the mixture was incubated at room temperature (RT) with periodic shaking overnight. After removing free drug with Sepharose CL-4B column, L-BORT was obtained.

Then, a post-insertion method was used to incorporate Tf into the above L-BORT. Briefly, holo (diferric) Tf in HEPES-buffered saline (HBS, pH 8, containing 5 mM EDTA) was reacted with 5× Traut's reagent to yield holo-Tf-SH. Free Traut's reagent was removed by dialysis using a MWCO 10,000 Dalton Float-A-Lyzer and against HBS. Holo-Tf-SH was coupled to micelles of Mal-PEG2000-DSPE at a protein-to-lipid molar ratio of 1:10. The resulting Tf-PEG2000-DSPE micelles were then incubated with the L-BORT for 1 hour at 37 °C at Tf-PEG2000-DSPE-to-L lipid ratio of 1:100. The resulting L-BORT and Tf-L-BORT were sterilized by filtration through a 0.22 µm membrane filter.

Characterization of the L-BORTs

Liposome size distribution was determined by dynamic light scattering on a NICOMP Submicron Particle Sizer Model 370 (NICOMP, Santa Barbara, CA). To determine the drug content, the liposomes were lysed by methanol and the BORT concentration was determined by absorption at 271 nm on a Shimadzu UV-visible spectrophotometer. Loading efficiency of BORT in liposomes was calculated based on the amount ratio of free and liposomal drugs, which were separated by the Sepharose CL-4B column.

Cellular uptake

Cellular uptake of R18-labelelled Tf-L-BORT or L-BORT was evaluated in K562 cells. For the studies, 4×10^5 cells were incubated with liposomes at 37 °C. After 1-hour incubation, the cells were then washed three times with PBS and observed on a Nikon fluorescence microscope (Nikon, Küsnacht, Switzerland) and measured by flow cytometry on a FACS Calibur flow cytometry (Becton Dickinson, Franklin Lakes, NJ).

Cytotoxicity

Cytotoxicity of BORT to K562 cells, or DOX combined with subtoxic BORT to K562/DOX cells, was evaluated by MTT cell proliferation assays (Promega, Madison, WI). To evaluate BORT toxicity, the cells were treated by different concentration of free BORT or L-BORT. At 48 hours after treatment, 20 µL of CellTiter 96® AQueous One Solution (Promega, Madison, WI) was added to each well and the cells were incubated at 37 °C for another 1 hour. Absorbance was measured at 492 nm on an automated plate reader (Molecular Devices, Sunnyvale, CA). To evaluate DOX toxicity, the K562/DOX cells were pre-treated with DOX for 24 hours and then subtoxic BORT formulation was added for additional 24-hour treatment. At this point, 20 µL of CellTiter 96® AQueous One Solution was added to each well and the cells were incubated at 37 °C for another 1 hour. Absorbance was measured at 492 nm on an automated plate reader.

Electrophoretic mobility shift assay (EMSA) probes

hBCR1-F 5’-GGGCCCCCCCGCCCTGTGCCCACGG-3’,

hBCR1-R 5’-CCGTGGGCACAGGGCGGGGGGG-3’;

hBCR1mut-F 5’-GGGCCCCTTTTTTCTGTGCCCACGG-3’,

hBCR1mut-R 5’-CCGTGGGCACAGAAAAAAGGGG-3’;

hBCR2-F 5’-GGGCCTCAGTTTCCCAAAAGGCACAGG-3’,

hBCR2-R 5’-CCTGTGCCTTTTGGGAAACTGAGG-3’.
